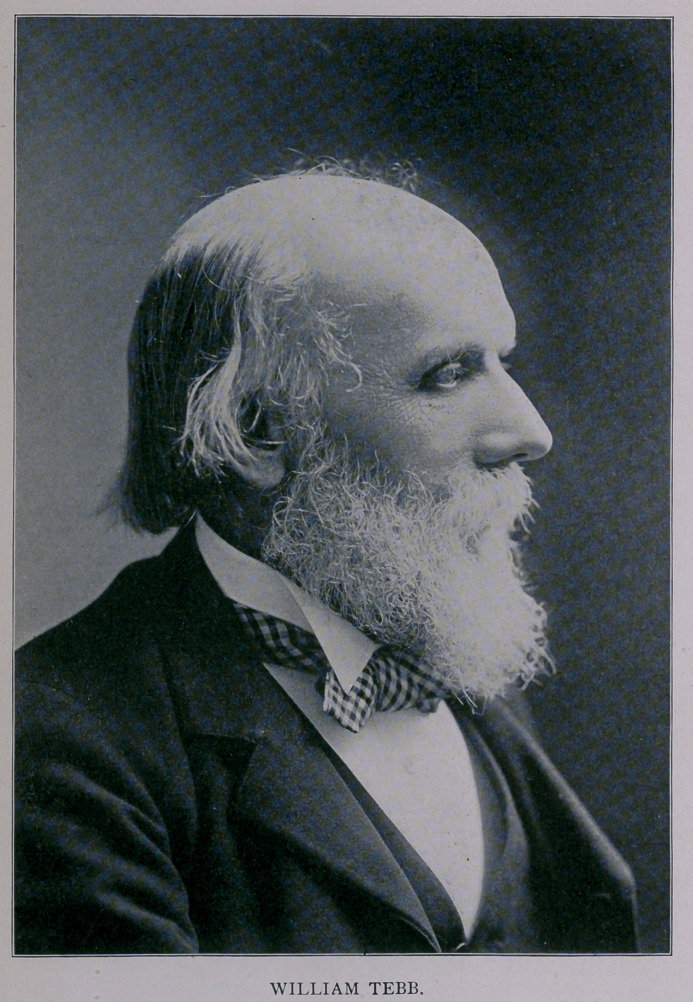# Introductory Memoir—Mr. William Tebb

**Published:** 1899-06

**Authors:** M. R. Leverson

**Affiliations:** Fort Hamilton, N. Y.


WILLIAM TEBB.

### INTRODUCTORY MEMOIR—MR. WILLIAM TEBB.

M. R. Leverson, M. D., Fort Hamilton, N. Y.

Whoever will take the pains to read the abstract here published of the extraordinary testimony given by Mr. William Tebb, before the Royal (British) Commission, on vaccination of 1889, will surely want to know something of the remarkable man by whom that testimony was given.

The portrait, which is here presented, was taken in 1896, and gives a fair idea of the man. The prevailing feature of the portrait, as in the character of the original, is evidently benevolence; but this is tempered, as his prominent eyebrows and broad and lofty forehead indicate, by keen observation and sound judgment.

The excellent business qualities, which the physiognomist and phrenologist will discern from the portrait, have enabled Mr. Tebb to acquire wealth, which his benevolence and combativeness have directed to a revolt against tyranny, and to channels of the highest value to mankind.

William Tebb belongs to a family of English yeomen, of the agricultural county of Westmoreland, noted for its beautiful scenery, and known as the home of the school of the Lake poets. In 1827, his parents removed to Manchester, where the subject of this memoir was born on the 22d of October, 1830. He inherited from his parents, and from a long-lived race of ancestors, a hardy constitution; but when a child he had an attack of scarlet fever and, in pursuance of the medical routine of the time, the little child, already enfeebled by his fever, was bled, both with leech and lancet. As a consequence of this *enlightened* medical practice of the nineteenth century, his constitution was impaired. He has suffered all his life subsequently from enfeebled digestion, and, for many years from excruciating nervous headaches on the least provocation.

Among his school-boy recollections is that of the boys flocking around the open surgeries every morning, watching the operation of bleeding as then practiced. The patients, many of them pale and sickly looking, needing more blood rather than to be depleted of that which they possessed, were by their venesection so weakened that, not seldom, they fainted under the operation.

It is well to bear in mind that the medical men of that day were well-nigh unanimous in asserting the benefits of periodical venesection. Professor Francis W. Newman, the celebrated author (brother of Cardinal Newman), told Mr. Tebb that at one time he had had seventy leeches applied to him, and that his constitution had been permanently weakened thereby.

The doctors in those days, at least in Lancashire, says Mr. Tebb, recommended bleeding in the spring and autumn; some of them insisted upon it every three months, and Mr. Tebb remembers his father telling him that in Westmoreland the doctors recommended bleeding once a month! To this the author of this sketch adds that his own father was bled and blistered to death under the direction of one of the most eminent physicians of the time in London, England. The medical belief in the efficacy of bleeding in fevers and in many other ailments (even in the case of accidents, when the patients had already lost blood) was almost unanimous both in England and on the continent of Europe, as well as in the United States. It is also worthy of note, that the doctors of that day were as arrogantly certain of their immense superiority over all previous generations of physicians as are the microbomaniacs of to-day of their superiority over the physicians of the last generation.

What would have been the consequences had parliament passed a law making the practice of venesection obligatory under pains and penalties, or even merely required that every child should be venesected as a condition of admission to the public schools, and with a staff of highly-paid officials to issue statistics in support of the practice, with a judicious system of bonuses for successful venesections, the issue of periodical reports, manifestoes, and ingeniously-concocted statistics by Boards of Health, together with appointments to offices, professorships, promotions, and membership in learned and influential associations?

The permanent physical injury Mr. Tebb sustained through the operation created in his mind a mistrust as to other medical practices, and particularly when it was found necessary to substitute force for reason, and to obtain their adoption with the aid of the police and the prison. Suffering under physical weakness from this treatment, he found himself handicapped in all athletic games and sports, but he took a vivid interest in watching them, and quietly admired the robust victors in a race or in a game of foot-ball.

Mr. Tebb received the amount of school education then general in the class of society in which he was reared, and which was about equivalent to what is now reached in the second or third grades of our grammar schools. It is to be observed that at that time there were but few free schools in England, except charity schools, and not many grammar schools. The public schools, such as Dulwich, Harrow, and Westminster, etc., had been perverted from the objects of their founders, and were, as they still are, monopolized by the rich. Consequently, all middle-class teaching in England had to be paid for by the parents, as was done by the parents of Mr. Tebb. The training and instruction given in such schools were by no means commensurate with their cost. Discipline was generally maintained by flogging.

When about fifteen, his father found him a place in the office of a leading business firm in his native city. The hours were long, and the duties irksome and monotonous, but they were lightened by looking forward to the evening classes of the Mechanics' Institution (though the mechanics who attended were, unfortunately, few in number), with its weekly lectures, conspicuous among which were those delivered by Ralph Waldo Emerson, of Concord, Mass.; by George Dawson, of Birmingham; James Crowden Clarke, and Henry Vincent. Then there were the Saturday evening concerts and entertainments given by the now octogenarian Henry Russell, author of "Cheer, Boys, Cheer," "To the West, to the West," "The Ship on Fire," "The Maniac," "Robin Ruff," etc.; Samuel Lover, author of *Handy Andy;* the inimitable John Parry; and the dramatic readings by John Kemble, Vandenhoff, and Bass. He took an active part in the Rusholme Young Men's Discussion Society, under the presidency of Mr. George Darling, and was appointed its secretary. Several papers, written by him, chiefly on social and literary subjects, were debated, but politics and religion were excluded. Being fond of traveling, he took frequent pedestrian excursions, walking through several counties of England, particularly Yorkshire, Lancashire, Nottinghamshire, and Leicestershire. He organized numerous excursions with his companions to places of historic or picturesque attractions, the most important of which was one to London in 1851, the year of the great international exhibition in Hyde Park and, after two weeks of unmixed enjoyment, the memory of which survives to this day (staying during the time with a relative in the metropolis), he planned an extension of the trip to Paris, whose magnificent palaces, art galleries, brilliant shops, boulevards and cafés, the gayety of the people, and their outdoor life completely enchanted him. Coming from a smoke-begrimed town, with blackened and unsightly factories, the brightness of the stately houses of Paris, and the clearness of its atmosphere was a revelation. When at St. Cloud, he saw Louis Napoleon, then Prince-President of France, who was at that time planning his notorious "coup d'etat" (coup de scélérat). The few days of his extended vacation passed all too quickly, but he returned home with indelibly pleasant reminiscences, and a growing impression that he should like to have a wider outlook on life and to see more of the world.

Mr. Tebb was young, in his "teens" when Cobden, Bright, Fox, Thompson, Elihu Burritt, Joseph Hume, and many others toiled to awaken the masses to a sense of the injustice of the "protective system," then dominant in Great Britain, as it still is, alas! in this country. He became interested in freedom of trade, and though too young to take an active part in that crusade for right, a lasting impression was made upon his mind, not only by the stirring speeches of the free-trade orators, but by the sights which met his eyes in the dens of misery, which were the abodes of many of the working classes. This misery was regarded by the free-trade orators as largely the result of "protection;" and it is true that this system of handing over to the idle the products of the labor of the industrious was one factor in that result; but, as has been clearly shown by the great man, who recently passed from among us, "protection" is but one form of that system of monopoly which is crushing the poor beneath the Juggernaut of special privilege, whereof the monopoly of the land is the corner-stone.

Mr. Tebb was a witness and, young as he was, an observant witness of the tremendous financial crisis of 1844, when the working classes of Manchester, in their terrible distress pillaged the bakeries to procure a momentary relief for their wives and children. Reduced to starvation wages, as are to-day so many of our own people, lodged in great numbers in miserable, windowless cellars, the death-rate among the working classes was appalling.

The evil was aggravated by the medical practice of venesection, then as much a medical fad in cases of sickness, as vaccination is to-day in the case of health, and, for that reason, somewhat less irrational. Fortunately for the human race, this sanguinary rite was never enforced or maintained by State law or State aid, as unhappily vaccination is to-day.

The confinement indoors, entailed by his occupation, was unfavorable to young Tebb's health; his prospects also did not seem encouraging to his youthful aspirations, and coupled with a desire to see more of the world, led him to seek a wider field in the United States. In August, 1852, he took passage in the Inman S. S. "City of Manchester," for Philadelphia. The passage was a stormy one—described by the captain as "an exceptionally rough passage," and lasted seventeen days, the decks being frequently covered with broken masts and torn sails. For nine days after his arrival no food would remain upon his stomach. Youth at length conquered, and he proceeded to deliver some of the numerous letters of introduction with which his friends had supplied him. He soon obtained employment in a wholesale dry goods store at the corner of John Street and Broadway, New York City, but finding it uncongenial to his tastes, he accepted an invitation to visit a friend near Hamilton, Canada West, whom he found living in a secluded district near the shore of Lake Ontario.

His friend, a graduate of Edinburgh University, was trying to perfect an industrial process, in which he promised young Tebb a share, and which, with the "bright hopefulness of youth," they both thought would lead to fortune.

The young inventors had no servant and very little furniture, and, as the younger, it fell to Tebb's lot to do most of the chores. Their food was of the simplest description—fruits and farinaceous articles, which with the keen, pure, and invigorating air of a Canadian winter, cutting wood, fetching supplies, excursions on the lake shore and to surrounding settlements, wonderfully improved his health.

The invention proved a failure, but on his return to New York, after an absence of about seven months, his friends scarcely recognized in the hearty young man before them the weakly dyspeptic of a few months past. A healthy environment, wholesome food, daily exercise, and hygienic advantages of many kinds had worked the change.

A friend at Lodi, N. J., was instrumental in procuring young Tebb the offer of an appointment as cashier in what was then the largest manufacturing establishment in Massachusetts, which he gladly accepted.

Slavery, its extension in the United States or its restriction and final abolition was the burning question of the day, and absorbed public attention in both Church and State.

It was not long before young Tebb was in the thick of the fight, writing to the local papers, speaking and taking every opportunity of visiting Boston, the headquarters of what Mr. W. H. Seward (Secretary of State under Mr. Lincoln's Administration) described as the "irrepressible conflict."

He became personally acquainted with William Lloyd Garrison, Wendell Phillips, Theodore Parker, Francis Jackson, Aden Ballou, and other anti-slavery apostles. The clergy, the professional, the wealthy, and so-called "respectable classes" were with few exceptions all on the side of the slaveholders, and cheerfully aided the authorities in the rendition of fugitive slaves to their masters. Marshall Rynders, of New York, with the aid of the police and firemen, cleared out an anti-slavery meeting held in that city as a public nuisance, in which disgraceful proceedings he was supported by nearly all the press. Theodore Parker declared that of 30,000 clergymen in the United States, he could count upon his ten fingers all who were openly opposed to slavery. Senator Hammond told Congress that he would yet call the roll of his thousand slaves from the top of Bunker's Hill. New territory was demanded from Congress for the extension of slavery. George Thompson, the famous English orator, whose stirring orations exercised so great an influence on the movement, had been mobbed and obliged to leave America. Garrison had been dragged through the streets of Boston with a rope round his neck. Charles Sumner had been bludgeoned in the Senate House at Washington. At Blackstone and neighborhood Mr. Tebb raised the question on all possible occasions, and held a discussion in the Millville Methodist Church, which was continued four nights, with a Democratic pro-slavery member of the Massachusetts Legislature, and obtained a majority vote condemning slavery. The minister of the Congregational Church at Blackstone, the Rev. T. E. Bliss, was an upholder of slavery, and the only occasion on which he would allow this question to be introduced in his meeting-house was to invite the Hon. Thomas H. Benton, Senator for Missouri, to deliver an address in defense of slavery and for the purpose of showing that the Constitution of the United States was favorable to the continuance of the domestic institution. The Rev. Mr. Bliss, in his Christian labors, went further, and occupied himself in persuading the Democratic proprietors of the Blackstone Manufacturing Company that persistent speaking against the enslavement of four millions of people on Sunday was an unpardonable form of "Sabbath breaking," an offense against religion and the best interests of the community. The Christian efforts of the Rev. T. E. Bliss were successful, and Mr. Tebb was requested to resign his appointment. Mr. Tebb was of opinion that the manager of the company, who wrote the letter requesting the resignation of Mr. Tebb, was ashamed of the business. A narrative of the incident was recorded in one of the local papers.

During his residence at Blackstone Mr. Tebb had the happiness to become acquainted with the lady who afterwards became his wife, Miss Mary Elizabeth Scott, of Scott Hill, Mass., then living at Hopedale, a community of anti-slavery and social reformers founded by Aden Ballou, a man honored and beloved by all for his elevated teachings and his spotless life. At a later period Mrs. Tebb was one of the first in their circle of acquaintances in London to perceive the absurdity of inoculating disease to obtain health, and assisted in organizing the Mothers' Anti-Vaccinating Society in London, which was the pioneer of the London Society for the Abolition of Compulsory Vaccination, and kept the movement alive after the death of Mr. Richard Gibbs; but we are anticipating.

## Figures and Tables

**Figure f1:**